# Supplementary material for: Failure to resolve inflammation contributes to juvenile onset cardiac damage in a mouse model of Duchenne muscular dystrophy
Source: Cell Death Dis. 2025 Jul 9;16(1):505. doi: 10.1038/s41419-025-07816-5 (PMC12241640; doi:10.1038/s41419-025-07816-5)
Supplement: Supplementary file 1 — Supplemental Data [file 41419_2025_7816_MOESM1_ESM.pdf]

Failure to resolve inflammation contributes to juvenile onset cardiac damage in a mouse model of Duchenne Muscular Dystrophy

James S. Novak[1,2†], Amy Lischin[1,3], Prech Uapinyoying[1,4], Ravi Hindupur[1], Young Jae Moon[1,5], Surajit Bhattacharya[1], Sarah Tiufekchiev-Grieco[1,6], Victoria Barone[1,3], Davi A. G. Mázala[1,7], Iteoluwakishi H. Gamu[1], Gabriela Walters[1], Jyoti K. Jaiswal[1,2†]

Supplemental Material.

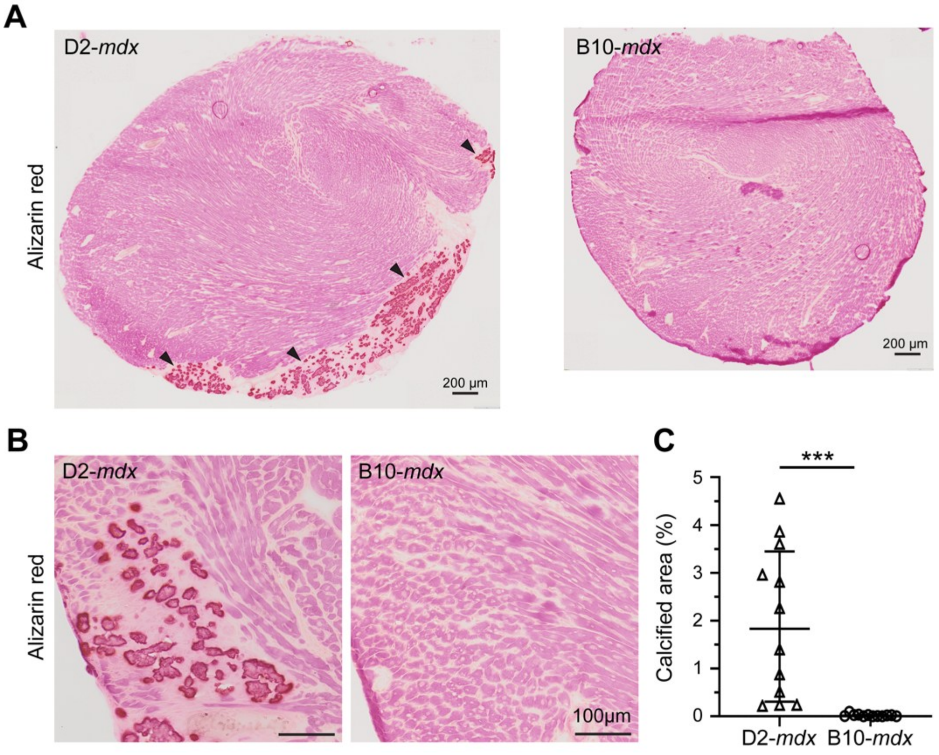

Supplemental Figure 1. Cardiac histopathology in juvenile D2-*mdx* model. A. Alizarin Red staining of juvenile D2-*mdx* and B10-*mdx* hearts (whole cross-section) showing right ventricular (RV) heart damage and calcification in juvenile D2-*mdx* hearts. Scale bars indicate 200µm (**A**). **B-C.** High magnification images from panel A, showing Alizarin Red staining of juvenile D2-*mdx* and B10-*mdx* hearts, and corresponding quantification of calcified fiber area per total tissue area. of fibrosis, damage and calcification are highlighted by black arrowheads (**C**). Scale bars indicate 100µm (**B**). Data represent median ± IQR. Statistical analyses performed using non-parametric Mann–Whitney test; ***$p < 0.001$.

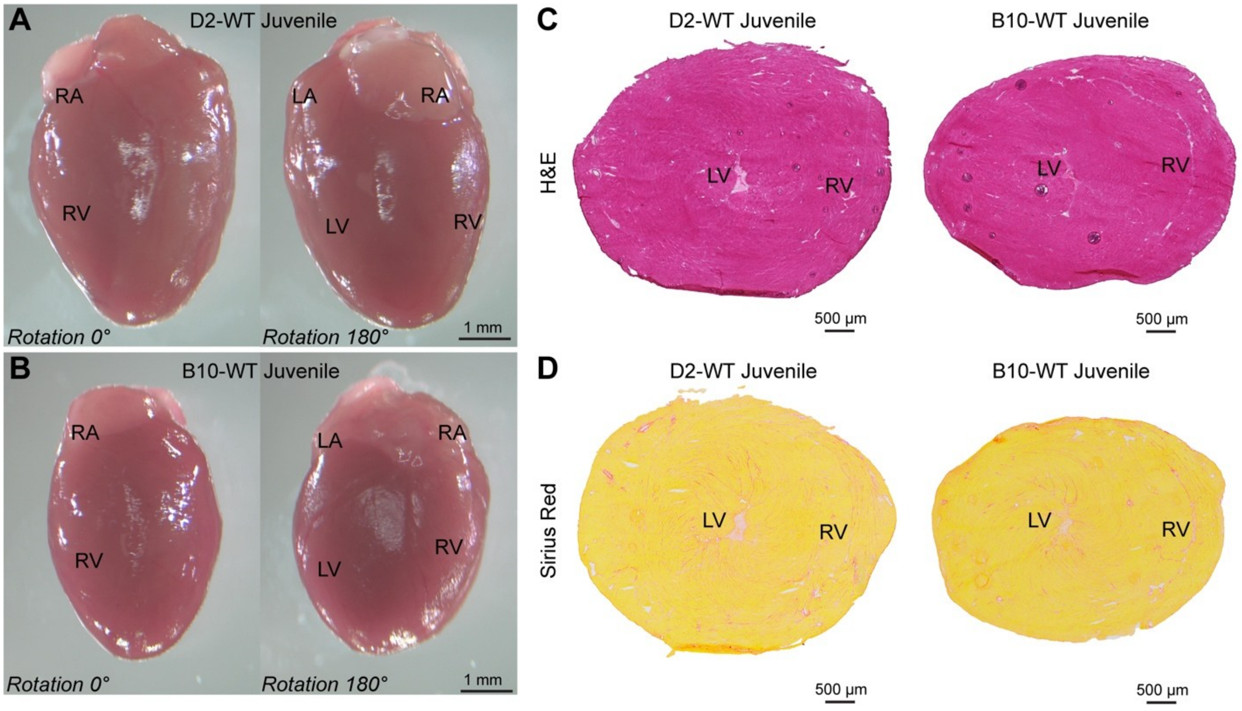

Supplemental Figure 2. Histopathological assessment of juvenile D2-WT and B10-WT hearts. Images show hearts harvested from juvenile (6 ± 0.5 wk) D2-WT and B10-WT mice. **A-B.** Whole tissue images with matched orientation of D2-WT (**A**) and B10-WT (**B**) hearts showing lack of any ventricular and atrial fibro-calcified damage. Scale bars indicate 1mm (**A-B**). **C-D.** Cross-sectional images of juvenile D2-*mdx* and B10-*mdx* hearts through the ventricular lumen, stained for histological features by H&E (**C**), and for fibrosis by Sirius Red (**D**). Scale bars indicate 500µm (**C-D**). Both D2-WT and B10-WT hearts lack any signs of fibro-calcified damage.

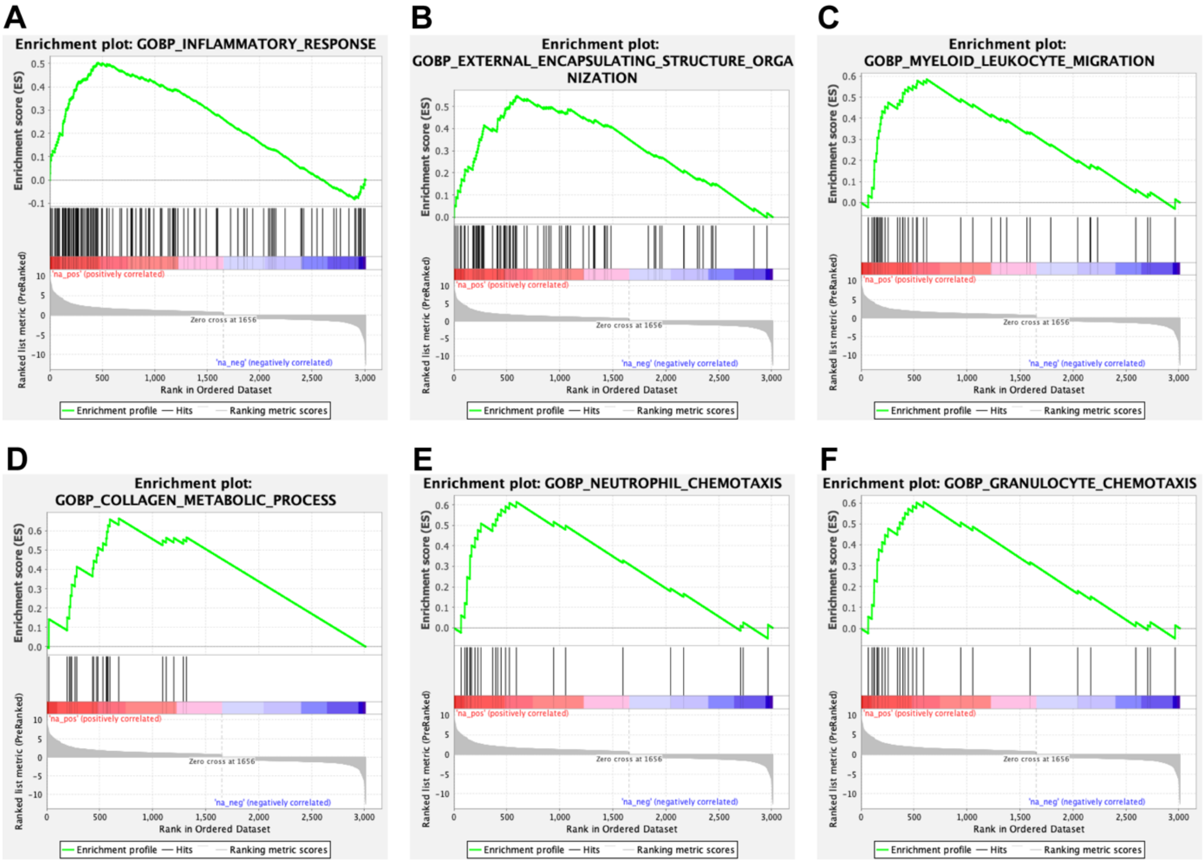

Supplemental Figure 3. GSEA enrichment plot analysis of top gene ontology (GO) hits from differential gene expression analysis in juvenile D2-*mdx* and B10-*mdx* hearts. A-F. Enrichment plot analysis of top immune-related (**A, C, E, F**) and extracellular matrix-related (**B, D**) GO biological process (BP) terms obtained using GSEA, including Inflammatory Response (**A**), Encapsulating Structure Organization (**B**), Myeloid Leukocyte Migration (**C**), Collagen Metabolic Process (**D**), Neutrophil Chemotaxis (**E**), and Granulocyte Chemotaxis (**F**).

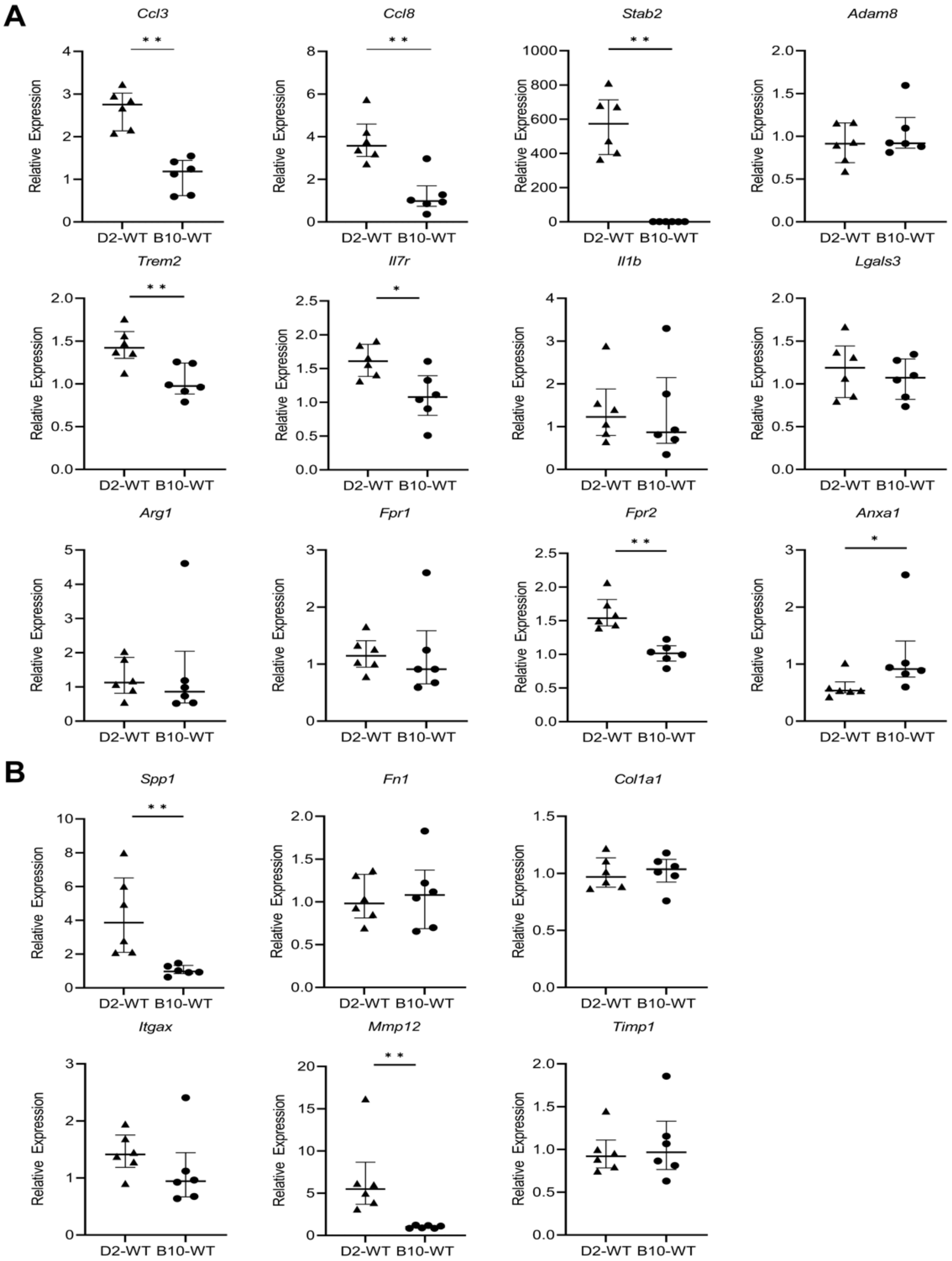

Supplemental Figure 4. Gene expression analysis of D2-WT and B10-WT hearts for selected immune and extracellular matrix targets. A. Relative gene expression analysis for dysregulated immune-related genes between D2-*mdx* vs. B10-*mdx* (**Figure 3**) assessed in juvenile D2-WT and B10-WT hearts. **B.** Relative gene expression analysis for dysregulated ECM-related genes between D2-*mdx* vs. B10-*mdx* (**Figure 4**) assessed in juvenile D2-WT and B10-WT hearts. Relative gene expression values normalized to internal *Hprt* transcript levels. Data represent median ± IQR. Statistical analyses performed using non-parametric Mann–Whitney test; $^{*}p < 0.05$, $^{**}p < 0.01$.
